# Effects of resistance exercise training on oxygen consumption and minute by minute efficiency during the six-minute walk test in female breast cancer survivors

**DOI:** 10.3389/fphys.2025.1694076

**Published:** 2025-12-01

**Authors:** Rodrigo Muñoz-Cofre, Macarena Artigas-Arias, Fernando Valenzuela-Aedo, Máximo Escobar-Cabello, Mariano del Sol, Daniel Conei, Jorge Sapunar, Rui Curi, Gabriel Nasri Marzuca-Nassr

**Affiliations:** 1 Departamento de Medicina Interna, Facultad de Medicina, Universidad de La Frontera, Temuco, Chile; 2 Universidad de La Frontera, Posdoctorado en Ciencias Morfológicas, Facultad de Medicina, Temuco, Chile; 3 Universidad de La Frontera, Doctorado en Ciencias mención Biología Celular y Molecular Aplicada, Facultad de Ciencias Agropecuarias, Temuco, Chile; 4 Centro de Investigación E Innovación del Cáncer. Fundación Arturo López Pérez OECI Cancer Center, Santiago de Chile, Chile; 5 Escuela de Kinesiología, Facultad de Salud, Universidad Santo Tomás, Temuco, Chile; 6 Universidad de La Frontera, Departamento de Ciencias de la Rehabilitación, Facultad de Medicina, Temuco, Chile; 7 Universidad Católica del Maule, Departamento de Kinesiología, Facultad de Ciencias de la Salud, Talca, Chile; 8 Universidad de La Frontera, Centro de Estudios Morfológicos y Quirúrgicos, Temuco, Chile; 9 Departamento de Procesos Terapéuticos, Facultad de Ciencias de la Salud, Universidad Católica de Temuco, Temuco, Chile; 10 Interdisciplinary Post-Graduate Program in Health Sciences, Cruzeiro do Sul University, São Paulo, Brazil; 11 Escola Superior Do Instituto Butantan (ESIB), São Paulo, Brazil; 12 University of Marília, UNIMAR, Marília, Brazil

**Keywords:** resistance exercise training, six-minute walk test, breast cancer, ventilatory efficiency, oxygen consumption (VO_2_)

## Abstract

**Objective:**

To determine the effects of resistance exercise training (RET) on oxygen consumption and minute-by-minute efficiency during the Six-Minute Walk Test (6MWT) in healthy postmenopausal female breast cancer survivors.

**Methods:**

Eleven healthy postmenopausal women, all breast cancer survivors, participated in a 12-week progressive RET program consisting of 36 training sessions. Sessions were conducted three times per week on nonconsecutive days and focused on both upper and lower limbs. Training loads ranged from 60% to 80% of participants’ one-repetition maximum (1RM). Before and after the 12-week program, participants underwent the 6MWT using a PNOE® metabolic analyzer. In addition to walking distance, oxygen consumption (VO_2_) and carbon dioxide output (VCO_2_) were assessed as key variables.

**Results:**

Following the intervention, the distance covered in the 6MWT significantly increased from 572.9 ± 62.6 m to 604.7 ± 39.9 m (p < 0.001). Work performed during the 6MWT (6MWORK) also increased significantly from 38415 ± 5611 to 40883 ± 5262 (p = 0.001). No significant differences were observed in relative VO_2_ (p = 0.116) or absolute VCO_2_ (p = 0.111). However, minute-by-minute VO_2_ analysis revealed a significant decrease in oxygen consumption during minutes 4, 5, and 6 of the 6MWT (28.91 ± 5.85 to 25.88 ± 5.61, p = 0.030; 32.08 ± 6.32 to 28.37 ± 5.40, p = 0.032; and 32.46 ± 6.57 to 28.60 ± 5.61, p = 0.045, respectively).

**Conclusion:**

Following the RET program as a single-arm pre- and post-intervention, a significant decrease in submaximal VO_2_ was observed during the second half of the 6MWT, specifically between minutes 4 and 6, accompanied by an increase in submaximal VCO_2_, which may have contributed to the significant improvements in 6MWT and 6MWORK distance.

## 1 Introduction

In recent decades, advances in early diagnosis and personalized treatments for breast cancer have increased significantly (Bray et al., 2024; Zhai et al., 2023). However, postmenopausal breast cancer survivors face a higher risk of musculoskeletal deconditioning (Smoot et al., 2009), attributable to factors such as prolonged physical inactivity and antineoplastic treatments, which negatively impact their functional capacity and quality of life (Zhang et al., 2020; Kubo et al., 2017). Specifically, in skeletal muscle, reductions in muscle fiber cross-sectional area, alterations in protein balance, and mitochondrial dysfunction have been reported, all of which compromise the maintenance of physical function (Artigas-Arias et al., 2024a).

In this context, physical training programs, particularly resistance exercise training (RET), have been shown to improve efficiency in activities of daily living (Vikmoen and Rønnestad, 2021; Vikmoen et al., 2016). A recent study in untrained young men demonstrated that RET reduces oxygen consumption during submaximal exercise, thereby enhancing work efficiency (Barrett-O’Keefe et al., 2012). The findings suggest that this improvement in efficiency is attributable to specific adaptations in the trained muscles: (i) changes in muscle fiber composition, (ii) enhancements in metabolic pathways, and (iii) improved neuromuscular coordination (Barrett-O’Keefe et al., 2012).

On the other hand, the six-minute walk test (6MWT) is used to assess exercise capacity and the cardiopulmonary response to movement beyond habitual activity, as well as to determine the response to medical treatments and/or physical interventions (ATS Committee on Proficiency Standa rds for Clinical Pulmonary Function Laboratories, 2002). Although the 6MWT is considered predominantly aerobic, its performance is also influenced by musculoskeletal factors such as muscle strength and endurance, particularly in populations with comorbidities or deconditioned states (Battaglini et al., 2009). Specifically, in female breast cancer survivors, adverse treatment effects (e.g., chemotherapy, radiotherapy, or hormone therapy) may result in reduced mobility and exercise tolerance (Bigman and Boright, 2021; Courneya et al., 2007). In this context, variables such as walking distance (WD) and lower-limb fatigue provide insights into musculoskeletal system performance during and after the test.

Although the 6MWT primarily assesses aerobic capacity, several studies do not incorporate minute-by-minute monitoring during the test, thereby overlooking systemic adjustments that may help explain changes in walking distance (6MWD). In 2001, Escobar et al. reported the temporal behavior of physiological and perceptual variables in response to different stimuli during the 6MWT. Their main findings indicated that continuous stimulation impacts 6MWD by increasing expenditure on a minute-to-minute basis (Escobar et al., 2001). However, the outcome also depends on the level of peripheral muscle strength, particularly in the lower limbs, which enables the maintenance of an efficient gait pattern while increasing the energetic cost of locomotion (Escobar et al., 2001; Medina et al., 2016). Thus, RET emerges as a strategy capable of improving work efficiency (Smoot et al., 2009; Kubo et al., 2017), reducing peripheral fatigue, and optimizing movement economy along submaximal activities such as the 6MWT.

Several studies have shown that resistance exercise training can improve exercise tolerance by enhancing neuromuscular efficiency, motor unit recruitment, and local fatigue resistance (Neil-Sztramko et al., 2017; Winters-Stone et al., 2012). In this regard, a stronger muscle requires a lower percentage of its maximal capacity to perform a submaximal task (Winters-Stone et al., 2012), such as walking, which translates into a lower perception of effort and greater functional capacity sustained over time.

Evidence on how strength training may influence aerobic function as measured by tests such as the 6MWT not only through cardiorespiratory effects but also via musculoskeletal, metabolic, and functional improvements, suggests that RET may exert beneficial effects on indirect cardiorespiratory parameters such as submaximal oxygen consumption and the anaerobic threshold. These adaptations could be reflected in an improved response along tests such as the 6MWT.

Previous evidence from our research group has shown that RET can improve work efficiency and reduce oxygen consumption during submaximal tasks in postmenopausal healthy and women breast cancer survivors (Artigas-Arias et al.). However, the underlying temporal behavior of these physiological adjustments throughout the 6MWT remains unexplored.

Therefore, the hypothesis is justified that RET, although not strictly an aerobic intervention may induce improvements in functional cardiorespiratory efficiency along prolonged walking tasks in postmenopausal breast cancer survivors. Accordingly, the present study aims to determine the effects of RET on oxygen consumption and work efficiency on a minute-by-minute basis along the 6MWT, in order to provide evidence of the functional benefits of this therapeutic approach in this population.

## 2 Methods

### 2.1 Participants

Eleven participants were included, consisting of healthy postmenopausal women who were breast cancer survivors (54 ± 3 years, BMI 26.6 ± 2.7 kg/m^2^). This study is part of a larger project designed to compare the effects of a 12-week progressive RET program on skeletal muscle mass, muscle strength, and physical performance in postmenopausal breast cancer survivors and already has previous publications (Artigas-Arias et al., 2025; Artigas-Arias et al., 2024b). The study was approved by the Scientific Ethics Committee of the Universidad de La Frontera, Temuco, Chile (registration n^o^. 004_23), in accordance with the Declaration of Helsinki. Written informed consent was obtained from all participants.

Inclusion criteria were as follows: (i) female participants, (ii) breast cancer survivors without recurrent disease, (iii) completion of adjuvant antineoplastic treatment ≥6 months prior to enrollment and at least 1 year since breast surgery (participants in the study had undergone surgery on average 4 ± 4 years earlier), (iv) age between 45 and 59 years, (v) BMI between 18.5 and 30 kg/m^2^, (vi) willingness to participate in the study, and (vii) No participation in a regular resistance training program during the previous 6 months.

Prior to enrollment, participants received a detailed explanation of the study, underwent a routine medical examination, and completed a general health questionnaire to ensure eligibility. Exclusion criteria were as follows: (i) cardiovascular diseases incompatible with physical exercise, (ii) comorbidities affecting body mobility, and (iii) smoking, use of nutritional supplements (leucine, glutamine, casein, whey protein, fatty acids, and creatine), and/or estrogen replacement therapy.

Participants completed a 12-week progressive RET program and were assessed in two separate testing sessions (test 1: PRE; test 2: POST), conducted 48 h before the first training session and 48 h after the final training session. Participants were instructed to refrain from alcohol consumption and from engaging in vigorous physical activity along the 2 days preceding each test.

### 2.2 Experimental design

In this single-arm, pre-post interventional report, participants underwent a comprehensive initial interview and received medical clearance to confirm their ability to complete the progressive RET program and the pre- and post-intervention assessments.

### 2.3 Blood pressure and heart rate

Prior to each 6MWT, blood pressure, resting heart rate (HR), and body weight were assessed. Blood pressure (BP) was measured using a digital OMRON® monitor (Japan) on the arm contralateral to the mastectomy or quadrantectomy. Heart rate was recorded using a POLAR® heart rate monitor (Polar® FS3, Kempele, Finland) (O’Brien et al., 2002).

### 2.4 Six-minute walk test with metabolic gas analysis

The 6MWT was performed following the guidelines of the American Thoracic Society (ATS) in a 30-m corridor, with standardized verbal encouragement provided throughout the test (Muñoz-Cofré et al., 2024). All measurements were conducted by an experienced evaluator. A PNOĒ metabolic analyzer (ENDO Medical, Palo Alto, CA), a portable and wireless device, was used to assess breath-by-breath oxygen exchange kinetics along the 6MWT (Artigas-Arias et al., 2025).

Heart rate was continuously monitored with a POLAR® ECG enabled chest strap (Polar® FS3, Kempele, Finland). Key respiratory variables including oxygen consumption (VO_2_), carbon dioxide output (VCO_2_), tidal volume, and respiratory rate were measured using a facemask equipped with a flow sensor and a gas analyzer, with a dead space of less than 70 mL to ensure minimal interference with gas-exchange accuracy. Participants carried data storage and transfer units secured in a dedicated harness. Real time breath by breath data were transmitted to a laptop, enabling continuous monitoring throughout the test. The additional equipment weight (approximately 800 g) was light enough not to affect the distance covered by participants. In addition, the modified Borg scale (1982) was used to quantify the subjective sensation of dyspnea (SSD) and fatigue (SSF), expressed on a 0–10 scale (Borg, 1982). Finally, the work performed along the 6MWT (6MWORK) was calculated as the product of body weight (kg)* walking distance in the 6MWT (Carter et al., 2003).

### 2.5 Resistance exercise training

Participants underwent a 12-week progressive RET program consisting of 36 individually supervised training sessions. Training sessions were conducted three times per week on non-consecutive days (Monday, Wednesday, and Friday), beginning with a warm up on a stationary bike for 5 min, followed by five sets of lower limb exercises using leg press and leg extension machines (TuffStuff Fitness International, California, United States). For the upper body, participants performed three sets of chest press, three sets of triceps extension, and three sets of seated row exercises (Fit Tech, Portugal). Passive rest intervals between sets were 2 min. The cool-down phase consisted of 3 large muscle group flexibility exercises (3 sets of 30 s hold each). The training sessions were conducted in groups of 2–4 participants.

The progression of the RET was based on the training load started at 60% of the participants’ one-repetition maximum (1RM) until reached 80% of 1RM. The 1RM was reassessed along the sixth week of the intervention to adjust training loads, as previously reported (Marzuca-Nassr et al., 2024).

### 2.6 Statistical analysis

Data were normally distributed according to the Shapiro–Wilk test and are therefore presented as mean ± standard deviation (SD). To assess changes in variables before and after the RET program, either a paired Student’s t-test or the Wilcoxon signed-rank test was applied, depending on data distribution. The same approach (Student’s t-test or Wilcoxon test) was used to compare HR, VO_2,_ and VCO_2_ before and after RET. The effect size (ES) was determined using Cohen’s d test using G*Power 3.1.9.4 software. It was defined as follows: an effect size <0.2 indicates no effect; 0.2–0.49 indicates a small effect; 0.5–0.79 indicates a medium effect; and 0.8 indicates a large effect. Statistical significance was set at p < 0.05. All statistical analyses were performed using SPSS software (IBM SPSS Statistics, version 21), and figures were created with GraphPad Prism 8.2 (San Diego, CA).

## 3 Results

The baseline characteristics of the participants are presented in Table 1, and detailed clinical and surgical information has been published previously (Artigas-Arias et al., 2025; Artigas-Arias et al., 2024b). Specifically, mean age was 52 ± 5 years, body weight 67.1 ± 7.2 kg, height 158 ± 7.2 cm, and BMI 26.8 ± 2.1 kg/m^2^. At baseline, HR was 73.3 ± 6.3 bpm, systolic blood pressure (SBP) 114.2 ± 9.8 mmHg, and diastolic blood pressure (DBP) 78.5 ± 7.1 mmHg. Regarding the 6MWT, the distance walked was 572.9 ± 62.6 m, with a VO_2_ of 29.8 ± 8.3 mL·kg^−1^·min^−1^ (absolute VO_2_: 1.9 ± 0.5 L·min^−1^), and a VCO_2_ of 1.8 ± 0.5 L·min^−1^. All participants successfully completed both the 6MWT protocol and the resistance training sessions without complications.

**TABLE 1:** Participants characteristics.

Variable	BCS
Age (y)	52 ± 5
Weight (kg)	67.1 ± 7.2
Height (m)	158 ± 7.2
BMI (kg^.^m^2^)	26.8 ± 2.1
HR (bpm)	73.3 ± 6.3
SBP (mm Hg)	114.2 ± 9.8
DBP (mm Hg)	78.5 ± 7.1
6MWT (distance, m)	572.9 ± 62.6
VO_2_ (mL·kg^−1^·min^−1^)	29.8 ± 8.3
VO_2_ (L· min^−1^)	1.9 ± 0.5
VCO_2_ (L· min^−1^)	1.8 ± 0.5
Peak work rate (W)	91.6 ± 13
VE (L/min)	62.7 ± 11.8
RER	0.97 ± 0.09

Data presented as mean ± SD. BCS, postmenopausal breast cancer survivors; BMI, body mass index; HR, heart rate; SBP, systolic blood pressure; DBP, diastolic blood pressure; 6MWT, six-minute walk test; VE, minute ventilation; RER, respiratory exchange ratio.

RET adherence was systematically monitored throughout the intervention. Attendance was recorded for each training session, and individualized logs were maintained to document the prescribed and completed number of sets, repetitions, and loads for each exercise. For the present analysis, participants were included if they attended at least 80% of the total sessions (i.e., 36 sessions). On average, participants attended 96% of all planned sessions, indicating a high adherence rate. No adjustments in training volume were required during the intervention, as all participants were able to complete the prescribed workload. This monitoring procedure ensured accurate quantification of adherence and supported the feasibility and fidelity of the training intervention.

**FIGURE 1:**
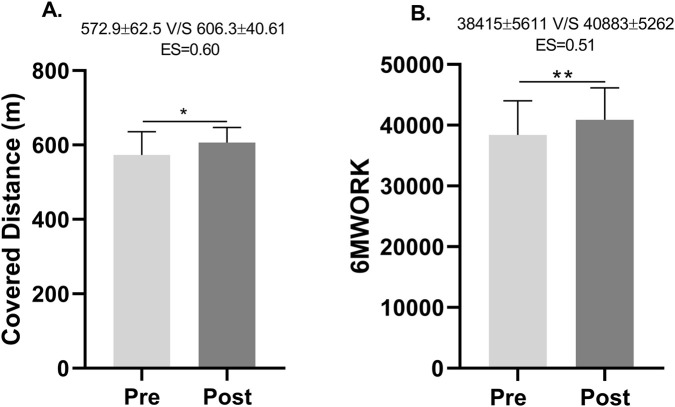
Distance covered and work performed along the Six-Minute Walk Test. ES, Effect size. **(A)** Distance covered in the Six-Minute Walk Test before and after the training program. **(B)** Work performed in the Six-Minute Walk Test before and after the training program.

Following the intervention, the 6MWT distance significantly increased from 572.9 ± 62.6 to 604.7 ± 39.9 m (p < 0.001), with an ES = 0.60. Similarly, 6MWORK significantly increased from 38415 ± 5611 to 40883 ± 5262 (p = 0.001), with an ES = 0.51 (Figure 1).

No significant differences were observed in relative VO_2_ (p = 0.116) or absolute VCO_2_ (p = 0.111). Minute ventilation (VE), however, significantly increased from 62.7 ± 11.8 to 67.2 ± 13.2 L/min (p = 0.043). Finally, the respiratory exchange ratio (RER) increased significantly from 0.97 ± 0.09 to 1.06 ± 0.06 (p < 0.001).

**FIGURE 2:**
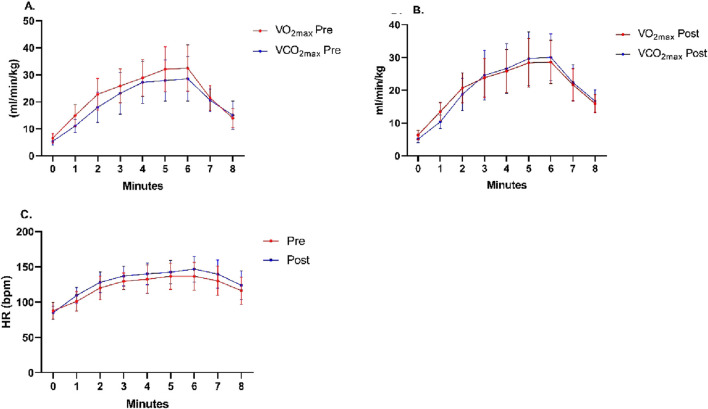
Minute-by-minute behavior of physiological variables along the Six-Minute Walk Test. **(A,B)** Oxygen uptake and carbon dioxide production along oxygen uptake and carbon dioxide production before and after the training program. **(C)** Heart rate along the test before and after the training program.

Comparing minute-by-minute VO_2_ in the 6MWT before and after RET, there was a significant decrease in oxygen consumption from 28.91 ± 5.85 to 25.88 ± 5.61 (p = 0.030), from 32.08 ± 6.32 to 28.37 ± 5.40 (p = 0.032), and from 32.46 ± 6.57 to 28.60 ± 5.61 (p = 0.045) at minutes 4, 5, and 6, respectively (Table 2) (Figure 2).

**TABLE 2:** Oxygen consumption, carbon dioxide production, and heart rate measured minute by minute along the six-minute walk test (6MWT).

Min	VO_2_		p	VCO_2_		p	HR		p
	Pre	Post		Pre	Post		Pre	Post	
0	6.50 ± 1.71	6.36 ± 1.45	0.840	5.40 ± 1.38	5.14 ± 1.04	0.610	87.73 ± 11.84	84.99 ± 9.37	0.494
1	14.94 ± 4.03	13.48 ± 2.81	0.387	11.08 ± 2.42	10.37 ± 2.05	0.551	101 ± 13.81	109.5 ± 11.2	0.128
2	22.81 ± 5.82	20.7 ± 4.55	0.264	17.96 ± 5.63	18.78 ± 4.93	0.764^W^	120.1 ± 16.75	128.1 ± 14.71	0.224^W^
3	25.89 ± 6.3	23.86 ± 5.93	0.225	23.13 ± 7.77	24.62 ± 7.58	0.399	129.5 ± 11.98	136.9 ± 13.77	0.201^W^
4	28.91 ± 5.85	25.88 ± 5.61	**0.030**	27.24 ± 7.81	26.65 ± 7.65	0.762^W^	132.4 ± 19.95	140 ± 15.4	0.194^W^
5	32.08 ± 6.32	28.37 ± 5.4	**0.032** ^**W**^	27.88 ± 7.69	29.68 ± 8.17	0.320	136.6 ± 18.53	142.4 ± 16.76	0.353
6	32.46 ± 6.57	28.6 ± 5.61	**0.045** ^**W**^	28.55 ± 8.26	30.07 ± 7.1	0.519^W^	136.6 ± 19.68	146.8 ± 18.27	0.190^W^
7	21.43 ± 4.59	21.69 ± 4.98	0.845	20.55 ± 4.18	22.38 ± 5.44	0.254	130.1 ± 20.85	139.7 ± 20.1	0.113
8	13.97 ± 3.56	14.95 ± 2.77	0.332	15.05 ± 5.28	16.7 ± 3.38	0.101^W^	116.2 ± 19.29	123.7 ± 20.37	0.247

Data presented as mean ± SD; Bold values denotes p < 0.05; VO_2_, oxygen consumption; VCO_2_, carbon dioxide output; HR, heart rate; w, Wilcoxon; Data were analyzed using paired Student’s t-test or the Wilcoxon signed-rank test.

## 4 Discussion

The aim of this study was to determine the effects of a 12-week progressive RET program on maximal oxygen consumption and work efficiency, assessed minute by minute along the 6MWT, in postmenopausal breast cancer survivors. In this regard, the main findings were: (i) a significant increase in the distance covered along the 6MWT after the training period, (ii) a significant increase in minute ventilation and RER following the intervention, and (iii) a significant reduction in VO_2_ consumption at minutes 4, 5, and 6 of the 6MWT after completion of the RET.

Following the training program, participants exhibited a significant increase in the distance covered along the 6MWT. This contrasts with the findings reported by Gorzelitz et al. (2022), who aimed to describe physiological and functional changes after a home-based strength training intervention. In their randomized controlled trial, they compared a 10-week home-based RET program with a waitlist control group. Their results showed no significant changes in the 6MWT (p = 0.29). This discrepancy could be due to the fact that in the present investigation: (i) the participants had a different type of cancer, (ii) on average they were younger in age, (iii) they received a greater number of sessions, and (iv) their training loads were adjusted during the intervention. (Gorzelitz et al., 2022).

In contrast, the present study was conducted through an onsite training program supervised by a physical therapist, which allowed for timely adjustments of workload throughout the intervention. This characteristic differs from the previously cited study, which relied on remote supervision. In this context, Martins et al. (2023) evaluated whether RET improves inflammatory markers, fatigue perception, fatigability, and physical performance in breast cancer survivors. Through a supervised and individually tailored strength training program, the authors reported a significant increase in the distance covered in the 6MWT (p < 0.001) in the intervention group (Martins et al., 2023), a finding consistent with the results of the present study. Therefore, this suggests that a resistance training program, when guided and adapted by a specialized professional, could lead to significant changes in the 6MWT. Schmidt et al. (2013) demonstrated high reliability in cancer patients when administering a single measurement of the 6MWT (ICC = 0.93; CV = 3%). The authors observed a learning effect of only 3.1% (∼17 m). Therefore, the effects observed here indicate that they are not due to familiarization with the 6MWT despite the lack of a control group (Schmidt et al., 2013).

The present study demonstrated a significant increase in both the distance covered along the 6MWT and in the outcome derived from the 6MWORK calculation (Figures 1A,B). This finding is consistent with the results reported by Carter et al. (2003), who evaluated the product of body weight and walking distance in 6 minutes (6MWORK) as a physiological outcome measure in patients with COPD. Their results showed that 6MWORK (body weight × distance walked along the 6MWT) averaged 35370 ± 9482 kg·m and 25643 ± 9080 kg·m for men and women, respectively (p < 0.0001). In addition, they observed that the correlation coefficient between VO_2_ and the 6MWT distance (r = 0.54; p < 0.0001) was lower compared to VO2 and 6MWORK (r = 0.81; p < 0.0001). Thus, while 29% of the variance in VO_2_ was explained by the distance covered in the 6MWT, 66% of the variance was accounted for when applying the 6MWORK calculation (Carter et al., 2003). Thus, the present study, in addition to demonstrating this significant increase in the distance traveled in the 6MWT and 6MWORK, adds a medium ES size after the RET.

With respect to the reported results, we can highlight the following after 12 weeks of RET: (i) the minute-by-minute behavior of VO_2_ along the 6MWT showed a steep increase up to minute 2; (ii) at minute 3, an inversion of the VO_2_ and VCO2 curves was observed, a phenomenon not present before the training intervention; and (iii) no statistically significant differences were found in heart rate before and after the 6MWT. This behavior is consistent with previous studies (Muñoz-Cofré et al., 2024; Hovington et al., 2009; Hovington et al., 2008), and suggests, on the one hand, that the physiological variables responded with an expected pattern under progressive workload. On the other hand, the intersection of the VO_2_ and VCO_2_ curves coincides with the significant increase in RER (Table 3), which suggests greater metabolic stress and a greater anaerobic contribution, despite the test being classified as submaximal and performed in medium-term breast cancer survivors. After 12 weeks of RET, both skeletal muscle and the cardiorespiratory system exhibited greater metabolic and ventilatory efficiency. In this context, a lower oxygen demand to perform the same workload—along with stable or slightly increased CO_2_ production—reflects a more efficient use of energy substrates and a more ventilatory efficiency response. Consequently, the crossover between VO_2_ and VCO_2_ becomes more evident or occurs earlier, indicating not a decline in performance but a favorable physiological adaptation to exertion (Deliceoğlu et al., 2024; Bauls et al., 2024).

**TABLE 3:** Metabolic and respiratory parameters along the 6MWT before and after 12 weeks of resistance exercise training.

	Pre	Post	p value
6MWT (meters)	572.9 ± 62.6	604.7 ± 39.9	**<0.001**
VO_2_ relative (mL/min/kg)	29.8 ± 8.3	28.4 ± 6.2	0.116
VCO_2_ absolute (L/min)	1.85 ± 0.5	1.97 ± 0.5	0.111
VE (L/min)	62.7 ± 11.8	67.2 ± 13.2	**0.043**
RER	0.97 ± 0.09	1.06 ± 0.06	**<0.001**
FAT (Kcal)	18.5 ± 10.1	10.6 ± 5.2	**<0.001**
FAT (%)	37.1 ± 21.1	19.5 ± 11.3	**<0.001**

Data presented as mean ± SD. Bold values denotes p < 0.05. 6MWT, six-minute walk test; VO_2_, oxygen consumption; VCO_2_, carbon dioxide output; VE, minute ventilation; RER, respiratory exchange ratio; FAT, Fat substrate. Data were analyzed using paired Student’s t-test.

Importantly, the reduction in oxygen consumption during the 6MWT was accompanied by an increase in work efficiency after RET, a finding previously reported by this same research group (Artigas-Arias et al.), further reinforcing the adaptive nature of this response. In breast cancer survivors, this ventilatory adjustment is particularly relevant, as resistance training may help mitigate respiratory fatigue and preserve muscle function compromised by oncologic treatments. Finally, the results after RET could indicate an improvement in neuromuscular control of expiration, a process that allows managing excess CO_2_ during submaximal exercise (Benítez-Muñoz et al., 2024; Vainshelboim et al., 2024; Beaver et al., 1986).

Other post-RET findings indicate that the crossover of the VO_2_ and VCO_2_ curves in the 6MWT could reflect improved walking economy or lower metabolic demand. These adaptations suggest that the respiratory system could be more efficient in managing the CO_2_, reinforcing the physiological benefits of individualized RET in cancer survivors (Oka et al., 2024; Rabinovich et al., 2004). Therefore, these variables should not be considered simply isolated physiological indicators, but rather as possible expressions of a reorganization through training, which improves exercise tolerance. Therefore, future studies should incorporate kinematic and neurophysiological analyses of the respiratory muscles to deepen the understanding of these phenomena.

This study has several limitations that should be acknowledged: (i) the inclusion of a control group would have provided stronger support for the conclusions. This situation means that the observed changes cannot be unequivocally attributed to the intervention. (ii) Although physical activity data were not collected in this study, a previous publication from the same project indicated moderate-to-low activity levels before the intervention, as assessed by the International Physical Activity Questionnaire (IPAQ), a valid and widely used self-reported tool. And (iii) the number of participants was relatively small due to the specificity of the condition under investigation. In conclusion, the progressive RET program optimized submaximal VO_2_ along the latter half of the 6MWT. Specifically, a significant decrease in VO_2_ was observed between minutes 4 and 6, accompanied by an increase in submaximal VCO_2_. This physiological response could help explain the significant improvements observed in distance covered in the 6MWT and 6MWORK.

## 5 Conclusion

In conclusion, following the RET program, a significant decrease in submaximal VO_2_ was observed during the second half of the 6MWT, specifically between minutes 4 and 6, accompanied by an increase in submaximal VCO_2_, which may have contributed to the significant improvements in 6MWT and 6MWORK distance. Considering the limitations of this study, given that it employed a single-arm design with no control group, the observed changes can be attributed entirely to the intervention.

## Data Availability

The raw data supporting the conclusions of this article will be made available by the authors, without undue reservation.
